# Recruitment and Activation of the ULK1/Atg1 Kinase Complex in Selective Autophagy

**DOI:** 10.1016/j.jmb.2019.07.027

**Published:** 2020-01-03

**Authors:** Eleonora Turco, Dorotea Fracchiolla, Sascha Martens

**Affiliations:** Department of Biochemistry and Cell Biology, Max Perutz Labs, University of Vienna, Vienna BioCenter, Dr. Bohr-Gasse 9/5, 1030 Vienna, Austria

**Keywords:** signaling, protein kinase, autophagosome, quality control, cargo receptor, PI3Kc1, phosphatidylinositol 3-phosphate kinase complex 1, LIR, LC3-interacting region, OPTN, Optineurin, PAS, pre-autophagosomal structure, Cvt, cytoplasm-to-vacuole, ER, endoplasmic reticulum

## Abstract

Autophagy is a major cellular degradation pathway, which mediates the delivery of cytoplasmic cargo material into lysosomes. This is achieved by the specific sequestration of the cargo within double-membrane vesicles, the autophagosomes, which form *de novo* around this material. Autophagosome formation requires the action of a conserved set of factors, which act in hierarchical manner. The ULK1/Atg1 kinase complex is one of the most upstream acting components of the autophagy machinery. Here we discuss recent insights into the mechanisms of ULK1/Atg1 recruitment and activation at the cargo during selective autophagy. In particular, we will focus on the role of cargo receptors such as p62 and NDP52 during this process and discuss the emerging concept that cargo receptors act upstream of the autophagy machinery during cargo-induced selective autophagy.

## Highlights

• Selective autophagy mediates cellular homeostasis by delivering harmful material into lysosomes for degradation.
• The activity of the ULK1/Atg1 kinase complex is required to initiate the formation of autophagosomes, which capture the cargo material.
• In selective autophagy receptors act upstream of the autophagy machinery by recruiting the Atg1/ULK1 complex to the cargo.
• Autophagy receptors directly bind to the FIP200 subunit of the ULK1/Atg1 complex.
• Autophagosome formation during selective autophagy can occur despite mTORC1 signaling.

## Graphical abstract

**Figure:**
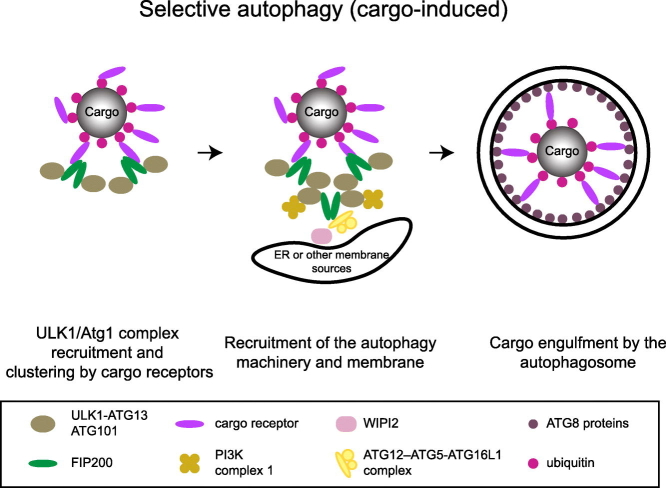

## Selective Autophagy

**Fig. 1:**
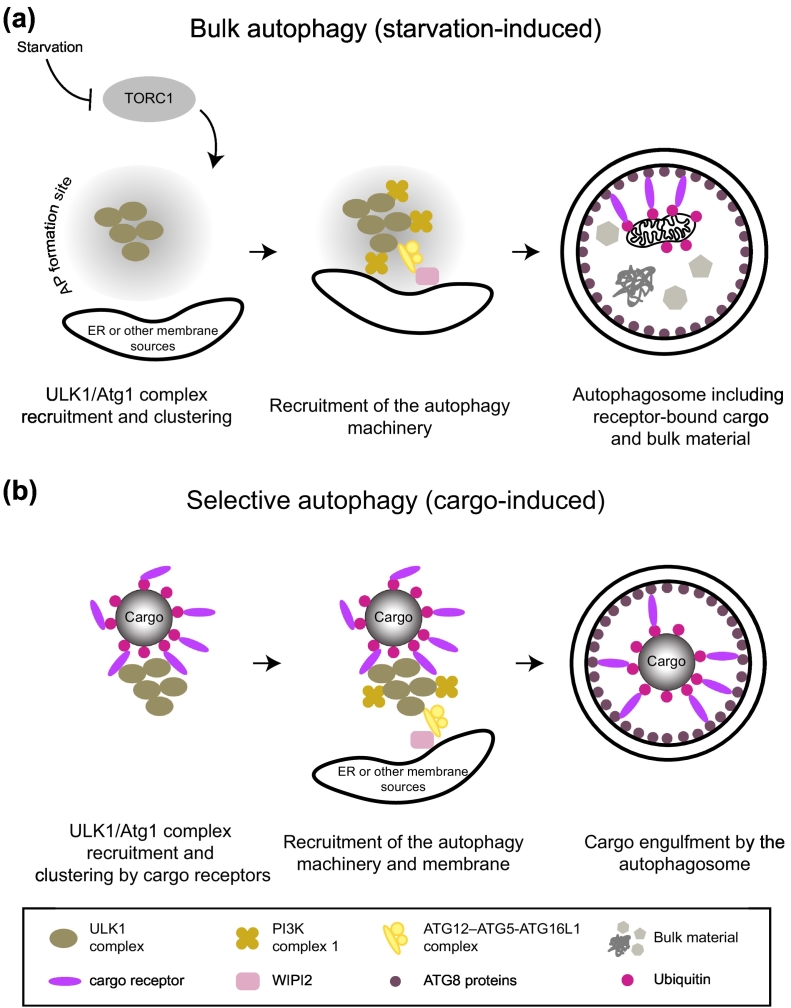
Mechanism of autophagy initiation during bulk (starvation-induced) and selective (cargo-induced) autophagy. (A) During starvation TORC1 is inhibited and the components of the ULK1/Atg1 complex become dephosphorylated. This event allows the assembly/recruitment of the ULK1/Atg1 complex at the site of autophagosome (AP) formation, in proximity of the ER or other membrane sources. ULK1/Atg1 clustering at the autophagosome formation site leads to its activation by auto-phosphorylation, which, in turn, triggers the recruitment of the autophagy machinery. This results in the formation of an autophagosome, which engulfs bulk material and receptor-bound cargo through the interaction of the receptor with ATG8-family proteins on the isolation membrane. (B) During selective autophagy, cytoplasmic cargo is recognized and bound by cargo receptors (directly or via ubiquitin), which induce autophagy by recruiting the ULK1/Atg1 complex to the cargo. Clustering of the ULK1/Atg1 complex at the cargo leads to its auto-activation and the recruitment of the autophagy machinery and membranes, which are necessary for the formation of an autophagosome, which subsequently specifically engulfs the cargo. A legend of shapes and colors used in the figure is shown below the panels.

Macroautophagy (hereafter autophagy) is a catabolic process wherein portions of the cytoplasm are enclosed by double-membrane vesicles, the autophagosomes, and delivered to the lysosomes for degradation. Autophagy is divided into non-selective (bulk) and selective processes (Fig. 1). The first is triggered by stresses such as the lack of nutrients and is regulated by the cellular nutrient sensor kinases AMPK and mTORC1 [1], [2], [3], [4], [5], [6], [7], [8]. Selective autophagy is a degradative pathway that can occur in the absence of starvation and has an important role in the maintenance of cellular homeostasis by specifically targeting cellular substances. Intracellular pathogens, protein aggregates, damaged organelles, ferritin, and peroxisomes are among the many substrates for selective autophagy [9]. It thereby protects from numerous diseases such as cancer, neurodegeneration, and uncontrolled infections and has also an important role in development [10], [11], [12], [13], [14], [15].

Autophagosome formation requires a set of conserved factors that act during all forms of autophagy. These factors are also referred to as core machinery and include the ULK1/Atg1 kinase complex, the class III phosphatidylinositol 3-phosphate kinase complex 1 (PI3Kc1) containing the ATG14 subunit, the WD-repeat proteins interacting with phosphoinositides (WIPIs), ATG2, and the ATG8 and ATG12 conjugation systems [16].

During selective autophagy, this core machinery acts in conjunction with cargo receptors to orchestrate the formation of autophagosomes that capture specific cargo material [17]. Cargo receptors selectively bind the cargo through dedicated domains and simultaneously bind the growing isolation membrane through LC3-interacting motif (LIR)-dependent interactions with ATG8-family proteins, which decorate the autophagosomal membrane, thus guaranteeing selectivity and the progression of the process [18], [19], [20]. It is also becoming increasingly clear that cargo receptors additionally recruit the autophagy machinery in order to promote autophagosome formation in the vicinity of the cargo material destined for degradation [21], [22], [23], [24], [25], [26], [27], [28] (Fig. 1).

Cells express a number of cargo receptors, which have, at least partly, overlapping functions in the degradation of various cargoes. This is due to the fact that many of them, including the most extensively studied mammalian cargo receptors SQSTM1/p62, NBR1, NDP52, and Optineurin (OPTN), recognize ubiquitinated cargo material indirectly via their ubiquitin binding domains [29].

For example, p62 cooperates with NDP52 and OPTN for the clearance of intracellular pathogens, and NDP52 and OPTN have partly overlapping functions in the degradation of damaged mitochondria [28], [30], [31], [32]. TAX1BP1, a paralog of NDP52, was shown to have a role in the autophagic clearance of intracellular pathogens and damaged mitochondria [28], [33].

Other cargo receptors appear to be more specifically dedicated to one target. For example, Bcl2‐L-13, the functional homolog of yeast Atg32, and Nix are important for mitophagy [34], [35]; NCOA4 mediates the autophagic degradation of ferritin [36], whereas numerous ER resident receptors such as CCPG1, FAM134B, and RTNL3L mediate the degradation of parts of the endoplasmic reticulum (ER; reviewed in Ref. [37]).

More recently, a class of so-called regulatory receptors has been characterized, the TRIM protein family, which acts in precision autophagy [22]. These alternative receptor molecules can directly bind to autophagic targets without the need for ubiquitin or galectin signals and can recruit the core autophagy machinery such as the ULK1 complex and PI3Kc1. Apparently, canonical receptors and TRIM proteins act in concert for the initiation of selective types of autophagy as they have been found to bind to each other and to co-localize in cells (reviewed in Ref. [38]).

## Composition and Function of the ULK1/Atg1 Complex

The ULK1/Atg1 protein kinase complex represents one of the essential core factors of the autophagy machinery and acts most upstream of the core autophagy machinery. In *Saccharomyces cerevisiae*, the Atg1 kinase subunit, which was originally identified as Apg1p (AutoPhagy protein 1) [39], is part of a complex including Atg13 and the Atg17‐Atg31‐Atg29 sub-complex [40], [41], [42], [43]. Atg11 is a further subunit of the Atg1 kinase complex, which is important for selective autophagy [44], [45]. The C-termini of *S. cerevisiae* Atg11 and mammalian FIP200 show sequence homology, and both bind to cargo receptors as further discussed below [23]. The rest of the two proteins do not display any apparent homology. The Atg1 protein encompasses a N-terminal kinase domain and a C-terminal EAT (Early Autophagy Targeting/Tethering) domain encompassing two tMIT (tandem Microtubule Interacting and Transport) domains (MIT1 and MIT2) that mediate its interaction with two corresponding MIM motifs [Mit Interacting Motif, MIM(N) and MIM(C)] in the intrinsically disordered region of Atg13 [46], [47]. In nutrient-rich conditions, when autophagy is inhibited, Atg13 is highly phosphorylated by TORC1 and its interactions with Atg1 and Atg17 are reduced [1], [43], [46], [48].

Upon inhibition of TORC1 by nutrient deprivation, Atg13 is dephosphorylated and its affinity for Atg1 and Atg17 is increased, resulting in Atg1 kinase complex formation, clustering at the pre-autophagosomal structure (PAS), which is the site of autophagosome formation in yeast, and Atg1 kinase activation [48]. Activation of the Atg1 kinase requires its auto-phosphorylation at Thr226 and Ser230 within its activation loop [49], [50]. At the PAS, the Atg1 kinase complex phosphorylates a number of factors required for the progression of autophagosome formation (reviewed in Ref. [40]). However, it also has non-catalytic functions in organizing the autophagy machinery and thus the progression of autophagy [41]. For example, the EAT domain in Atg1 is responsible for its association with highly curved membranes at least *in vitro* [42], while Atg13 contains an HORMA domain that mediates its interaction with Atg9 *in vivo* [51].

The human homolog of Atg1 is represented by the ULK1/2 proteins. Both kinases have somewhat redundant functions in the induction of autophagy [52], [53]. The ULK1 subunit was first described in *Caenorhabditis elegans* as UNC-51 (UNCoordinated-protein 51) [54] and later the mammalian counterpart ULK1 (Unc-51-like Kinase) was identified in mouse [55]. Similar to yeast Atg1, mammalian ULK1/2 also bear phosphorylation sites in their activation loop at Thr180, and this phosphorylation is necessary for their activation [56]. They are part of a protein complex composed of four subunits, which also includes ATG13, ATG101, and FIP200/RB1CC1 [5], [6], [7], [57], [58]. Mammalian ATG13 has only low sequence homology with yeast Atg13 [47], [59], but it also contains a HORMA domain in its N-terminus, through which it interacts with the ATG101 subunit that almost entirely consists of a HORMA domain [58], [60], [61]. ATG13 has also been shown to bind an LQFL motif (aa 582–585) in the FIP200 N-terminus, thus possibly acting as a bridge between the ULK1 and FIP200 subunits within the complex [62]. Similar to the yeast counterpart, ATG13 is highly phosphorylated under normal conditions and becomes partially de-phosphorylated upon induction of autophagy [5], [6], [7], [63]. However, in contrast to *S. cerevisiae*, the formation of the mammalian ULK1 complex including the ULK1-ATG13 subcomplex appears not to be regulated by nutrient availability [6].

In addition to regulating the kinase activity of ULK1, the subunits of the complex are platforms for the binding or indirect recruitment of downstream acting autophagy factors including the PI3Kc1 [64], [65] and possibly the WIPIs and DFCP1 [60], [61], as well as the ATG12–ATG5‐ATG16 complex [66], [67]. FIP200, ATG13, and ULK1 also bind to ATG8-family proteins that decorate the nascent autophagosomal membrane [68], [69]. In this manner, the ULK1 complex acts not only to activate the autophagy machinery by phosphorylation [5], [40], [41], [65], [70], [71], [72], [73] but also to spatially organize the factors and membranes for productive autophagosome formation. Particularly interesting in this regard is the FIP200 subunit. It is thought to be the functional counterpart of the *S. cerevisiae* Atg17 and Atg11 proteins, which function as scaffolds for the recruitment of the Atg1 kinase complex in starvation-induced bulk autophagy and selective autophagy, respectively [23], [25], [45], [57], [74], [75].

Most of the knowledge about the activation of the ULK1/Atg1 complex is derived from studies in nutrient starved cells where the cytoplasm becomes generally permissive for its activation by the downregulation of mTORC1 activity. In this review, we want to focus on the recruitment and activation of the ULK1/Atg1 complex in selective autophagy processes that can also occur in the presence of global inhibitory mTORC1 activity. This is still an only partially understood aspect, but recent studies have given us some insights into this process in mammalian cells, and we will therefore focus our discussion mainly on mammalian selective autophagy. However, pioneering studies of the cytoplasm-to-vacuole (Cvt) pathway, which in *S. cerevisiae* constitutively delivers the prApe1 peptidase and other enzymes into the vacuole, have uncovered the basic principles of selective autophagy. We will therefore briefly summarize these findings [76], [77].

## Selective Autophagy Initiation in Yeast

**Fig. 2:**
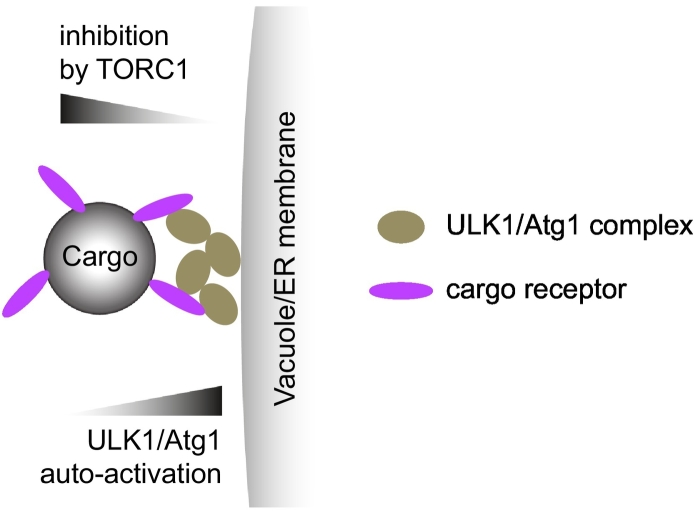
Possible mechanism of ULK1/Atg1 complex activation by clustering during selective autophagy. The ULK1/Atg1 complex is recruited and clustered by the cargo receptor and positioned between the cargo and the vacuole/ER membrane. Under this condition, ULK1/Atg1 molecules are protected by the inhibitory signals of TORC1 and at the same time are close enough to each other for auto-phosphorylation and thus activation.

Studies of the Cvt pathway in *S. cerevisiae* derived a model where Atg19 cargo receptor-bound prApe1 cargo particles recruit and activate the Atg1 kinase complex via the Atg11 scaffold protein. This activation can occur even under nutrient-rich conditions. The model was validated for other selective autophagy types in yeast including pexophagy [25], [44], [74], [78], [79], [80]. Clustering of the Atg1 kinase subunits on the cargo receptor-bound prApe1 via Atg11 ultimately allows for the auto-phosphorylation of the residue Thr226 in the activation loop of Atg1, which is necessary for kinase activity and leads to local kinase activation [25], [49], [50], [74]. Thus, in the presence of high TORC1 activity, the cargo may serve as a platform for the concentration of Atg1 shielding it from inhibitory phosphorylation by TORC1. This model was refined by the finding that efficient activation and, by implication clustering of Atg1 at the cargo, requires the Atg13-mediated targeting of Atg1 to the vacuole, which is the site where autophagosomes and Cvt vesicles form in *S. cerevisiae* [74]. Therefore, when shielded by two surfaces, the prApe1 cargo and the vacuolar membrane, Atg1 may be maximally protected from inhibitory signals while at the same time being highly concentrated, thus promoting autoactivation (Fig. 2).

## Cargo Receptor-Mediated Initiation of Selective Autophagy in Mammals

As with the yeast system, most studies addressing the mechanisms of autophagosome formation in mammals were conducted in starved cells, which revealed that a spatially defined structure such as the perivacuolar PAS in *S. cerevisiae* does not seem to exists and that autophagosomes can seemingly form throughout the cytoplasm. Autophagosome formation occurs at discrete foci in which the components of the autophagy machinery co-localize [81]. Analogous to the situation in yeast, the components of the ULK1 complex are among the earliest and most upstream acting factors in this process [75], [82], [83] (Fig. 1). The biogenesis of autophagosomes requires lipids, and a variety of organelles have been implicated as membrane donors for this process (reviewed in Ref. [84]). However, it has become clear that the ER has a fundamental and evolutionary conserved role in providing a platform for the assembly of the autophagy machinery and possibly also as source of lipids for autophagosome formation [83], [84], [85], [86], [87], [88].

The general distinction between starvation-induced bulk autophagy and selective autophagy can be somewhat confusing because a certain degree of selectivity also exists in bulk autophagy (reviewed in Ref. [17]). We here refer to selective autophagy as cargo-induced autophagy, where autophagosome formation is induced by the presence of the cargo material and which can occur when mTORC1 signaling is not globally reduced (Fig. 1). Under these conditions, autophagy can be largely exclusive with regard to the cargo material that is encapsulated by autophagosomes, similar to what is observed for the Cvt pathway in *S. cerevisiae* [17], [89], [90]. In the classical model of mammalian selective autophagy, the cargo receptors act downstream of the autophagy machinery to tether the cargo to the nascent ATG8-family proteins decorated autophagosomal membrane. While this model may be correct in case of starvation-induced autophagy, where some cargoes are selectively degraded (reviewed in Ref. [17]), it has emerged in the past years that the cargo receptors act upstream of the autophagy machinery in cargo-induced selective autophagy. For PINK1/parkin-dependent mitophagy, it was observed that the NDP52 and OPTN receptors are required to recruit ULK1 and the autophagy machinery, including ATG8-family proteins, to damaged mitochondria [28] suggesting that, analogous to the Cvt pathway, the cargo is able to induce autophagosome formation via the cargo receptors. This further suggested that cargo receptors are not mere tethers that link the cargo to the autophagosomal membrane but additionally function to orchestrate the autophagy machinery. Recent studies have yielded important insights into how the mammalian cargo receptors recruit the ULK1 complex to the cargo and activate the autophagy machinery. Particularly important in this regard is the FIP200 subunit of the ULK1 complex.

Direct tethering of NDP52 to the surface of mitochondria was found to be sufficient for the recruitment of the autophagy machinery, including FIP200, ATG14, and ATG16L1 [24]. Ectopic localization of FIP200 or ULK1 to mitochondria induced mitophagy, suggesting that localization of the ULK1 complex to the cargo is sufficient for autophosphorylation and initiation of selective autophagy [24]. Activation of the ULK1 complex upon tethering to the cargo is dependent on the ULK1 kinase activity and resistant to mTORC1 signaling, since the ability of the ULK1 complex to induce mitophagy upon tethering to mitochondria is affected in cells treated with ULK1/2 inhibitors, but not by the overexpression of mTOR [24]. The recruitment of the ULK1 complex to mitochondria by NDP52 is based on the interaction between the NDP52 SKICH domain and a C-terminal leucine zipper domain in FIP200 [24].

**Fig. 3:**
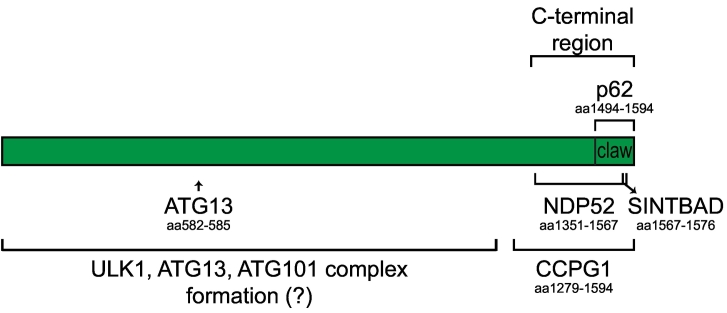
Schematic, scaled representation of FIP200 as scaffold for autophagy. The N-terminal and central region of the protein are involved in complex formation with ATG13, ATG101, and ULK1. In particular, ATG13 binds an LQFL motif (aa 582–585) in FIP200 N-terminus. The C-terminal region, including the claw domain (aa 1494–1594), is mainly involved in the interaction with cargo receptors. While NDP52 was shown to interact with a leucine zipper motif within the C-terminal region (1351–1567), the p62 binding pocket is located in FIP200 claw domain. It remains to be established if NDP52 could also bind the same p62 binding pocket and *vice versa.* The binding site for the ER-phagy receptor CCPG1 was also mapped to FIP200 C-terminal region (aa 1279–1594). Moreover, the FIP200 claw domain can also bind the TBK1 adaptor SINTBAD (aa 1567–1576).

A very similar situation is observed for NDP52-mediated autophagic clearance of cytosolic *Salmonella* Typhimurium [26]. Here, NDP52 recruits the ULK1 complex via FIP200 to the surface of the bacteria, and this recruitment is also based on the interaction of the NDP52 SKICH domain and a C-terminal region of FIP200 that includes the leucine zipper shown to be important for its recruitment to mitochondria (Fig. 3) [24], [26]. TAX1BP1 was also shown to interact with FIP200 in a direct manner [26]. Activation of antibacterial autophagy also required the interaction of NDP52 with the TBK1 kinase adaptor proteins SINDBAD/NAP1 as well as the binding of the very C-terminal domain of FIP200, named claw ([23], see below) to SINDBAD/NAP1 (Fig. 3), suggesting that ULK1 kinase activity and TBK1 activity must be spatially coupled for autophagosome formation [26]. Similarly, TBK1 activity is also required for induction of mitophagy [24], [91], [92].

Of note, the NDP52 paralog TAX1BP1 was shown to also mediate ferritinophagy through direct interaction with the NCOA4 receptor and TBK1. In this example, TAX1BP1 serves as an adaptor to bring together NCOA4‐ferritin complexes, the ULK1 complex, and TBK1 leading to the delivery of ferritin into the lysosome in a process independent of the ATG8 conjugation system [93].

Apart from NDP52, it was also recently shown that the cargo receptor p62 directly interacts with the very C-terminal region of FIP200 (Fig. 3) [23]. This region of FIP200 shows a high degree of homology to the C-terminus of yeast Atg11, which also interacts with cargo receptors [23], [94]. Structural studies showed that the C-terminal region of FIP200 includes a globular domain at its extreme end, which dimerizes and has the shape of a claw. The claw domain directly interacts with a central region of p62, which also contains its LIR motif [23]. When the LIR motif was mutated, the interaction of p62 with the FIP200 C-terminal region was severely decreased and the LIR alone was sufficient for the interaction with FIP200 claw. The binding site for the LIR motif in the claw was mapped to a hydrophobic pocket lined by positively charged residues [23]. In cells, the claw domain was shown to be important for the recruitment of FIP200 to condensates formed by p62 and ubiquitinated proteins [95], [96] and their autophagic degradation [23]. Interestingly, FIP200 was not essential for the recruitment of the ULK1 kinase to the condensates, although their autophagic degradation was abolished upon loss of FIP200 [23]. This suggests that redundant mechanisms for the recruitment of ULK1 to the p62‐ubiquitin condensates exist and further that FIP200 has roles beyond the mere recruitment of ULK1.

The ER-resident protein CCPG1 was recently identified as non-canonical cargo receptor for ER-phagy, the autophagic degradation of parts of the ER and its expression is induced by the unfolded protein response [27]. It was shown that CCPG1 binds ATG8-family proteins through a LIR motif and FIP200 through distinct motifs named FIP200-interacting regions (FIRs) [27]. Following this nomenclature, the same name was subsequently given to the FIP200-interacting region in p62 [23]. Analogous to NDP52 and p62, the interaction of CCPG1 with FIP200 also occurred via the C-terminal region of FIP200 (Fig. 3). The binding of CCPG1 to FIP200 is required for ER-phagy and for the incorporation of CCPG1 into autophagosomes [27].

Recently, it was shown that ULK1 and LC3B-II are co-immunoprecipitated by overexpressed Bcl2‐L‐13, a cargo receptor that recognizes damaged mitochondria. Bcl2‐L-13 may recruit LC3B to damaged mitochondria, where it interacts with the ULK1 LIR motif, leading to the recruitment of the ULK1 complex, whose members (ATG13, FIP200, and ATG101) are necessary for mitophagy [97]. Considering the recent evidence, it would not be surprising if Bcl2‐L‐13 was also able to recruit the ULK1 complex by direct binding to one of its subunits.

Thus, a paradigm has emerged from recent studies suggesting that cargo-driven selective autophagy is mediated by upstream acting cargo receptors, which recruit the ULK1/Atg1 kinase complex (Fig. 1) at least in part via the C-terminal region of FIP200 subunit to drive local formation of autophagosomes [23], [24], [26], [27]. However, there are also notable differences between the exact mechanisms of the FIP200 recruitment. While NDP52 was shown to interact with a C-terminal leucine zipper/coiled coil domain of FIP200 [24], [26], p62 was shown to interact with the extreme C-terminal claw domain of FIP200 [23]. It remains to be established if NDP52 can also bind the claw and if p62 is additionally able to interact with the coiled coil domain of FIP200. Furthermore, NDP52 and CCPG1 employ separate motifs for the interaction with FIP200 and ATG8-family proteins [24], [26], [27], whereas for p62, the FIP200 binding site and the LIR overlap and therefore the interactions of LC3B and the FIP200 C-terminal region with p62 are mutually exclusive [23]. The reason for these differences might result from their different cellular functions and in particular from the fact that their main cargoes are distinct. NDP52 acts in antibacterial autophagy and mitophagy [28], [32]. These events are rare but when they occur, the cell must act quickly to remove the harmful material and may therefore recruit the autophagy machinery with high affinity. Whether or not parts of the autophagy machinery, including FIP200, are degraded under these conditions may not be so relevant for the cell. In contrast, p62 acts to degrade ubiquitinated proteins in a constitutive manner ([98] and reviewed in Refs. [99], [100], [101]) and p62 is locally highly concentrated in the p62 and ubiquitin containing condensates [95], [96]. Therefore, the low affinity of the p62 – FIP200 interaction [23] may prevent sequestration of the autophagy machinery within the condensates. The fusion of the FIP200 and ATG8-family protein binding sites may allow the ATG8-family proteins to release FIP200 from the cargo as they become concentrated on the nascent autophagosomal membrane. Indeed, it was observed that FIP200 is not a major component of the autophagosomes containing p62 condensates and its release from the cargo was dependent on the activity of the ATG8 conjugation machinery [23].

An important question for the future is the precise mechanism of ULK1 activation at the cargo. It is likely that receptor/scaffold-mediated ULK1 clustering at the cargo triggers ULK1 autophosphorylation, similar to the yeast Atg1 activation mechanism during the Cvt pathway [25], [74]. Indeed, autophosphorylation of ULK1 was reported on T180 in the activation loop [102], and a T180A mutant of ULK1, unlike the wild-type protein, was unable to trigger mitophagy after artificial tethering to mitochondria [24].

Taken together, these studies provide mechanistic insights into how cargo recognition is coupled to autophagy initiation through direct interaction of cargo receptors with the scaffold protein FIP200.

## Other Scaffolds in the Initiation of Selective Autophagy

Other proteins apart from canonical cargo receptors were proposed to act as organizers of selective autophagy. Among them is Huntingtin. Huntingtin directly participates in cargo selection as it was shown to interact with the ULK1 complex and p62 in mammalian cells and with their respective homologs in *Drosophila melanogaster* [103], [104]. In particular, Huntingtin interacts with ULK1 and displaces it from mTORC1 to promote ULK1 activation. In addition, the binding of Huntingtin to p62 enhances the affinity of p62 for K63-linked ubiquitin chains and ATG8-family proteins [103]. Interestingly, both Huntingtin knockdown and its overexpression neither affect mTORC1 activity nor influence the association of AMPK and its phosphorylation of ULK1. Overexpression of Huntingtin is sufficient to enhance the formation of ULK1 – Huntingtin complexes and to antagonize the inhibitory effect of mTORC1 on ULK1. These data led the authors to conclude that Huntingtin might serve as a scaffold protein in selective autophagy [103].

ALFY is another example of a protein proposed as a scaffold for selective autophagy of ubiquitinated proteins and p62 containing condensates [105], [106], [107]. ALFY localizes in the nucleus and relocates to the cytoplasm during proteotoxic stress, where it directly binds p62, ATG5, and phosphatidylinositol-3-phosphate (PI3P) [106], [108]. In this way, ALFY could bring together the cargo, the ATG8 conjugation machinery, and the PI3P containing isolation membrane, possibly bypassing the ULK1 complex. Given the nuclear localization of ALFY in resting conditions, it is likely that its role in aggrephagy becomes important only in extreme stress situations, where it reinforce p62-dependent clearance of protein aggregates.

Other examples of proteins exist, which bring together cargo material and the autophagy machinery. For example, the TRIM20 and TRIM21 proteins interact with ULK1, Beclin 1, ATG16L1, and ATG8-family proteins to mediate the degradation of inflammasome components [22], [38], and TRIM5a binds to ULK1, Beclin1, and ATG8-family proteins to degrade retroviral capsid proteins [109]. Furthermore, TRIM16 was shown to recruit the autophagy machinery to damaged lysosomes via Galectin 3 [110].

## The Role of Phosphorylation in the Initiation of Selective Autophagy

The role of phosphorylation in autophagy is manifold and has been extensively studied in starvation-induced autophagy as well as selective autophagy (reviewed in Refs. [8], [40]). With respect to the phosphorylation mediated control of ULK1/Atg1 recruitment and activation, phosphorylation has been shown to enhance cargo receptor recruitment to the cargo and also to increase the affinity of the receptors for the ULK1/Atg1 complex.

Regarding the phosphorylation-dependent recruitment of cargo receptors to the cargo, phosphorylation of S403 in p62 ubiquitin binding (UBA) domain by casein kinase 2 (CK2) and TBK1 increases its affinity for ubiquitinated cargo, thereby enhancing its autophagic clearance [111], [112], [113]. It was also shown that p62 is phosphorylated at S409 by ULK1, and it was proposed that this post-translational modification would be necessary for the subsequent phosphorylation of S403 described above [114]. In addition, the OPTN UBAN domain is phosphorylated by TBK1 at S473 and S513, which results in enhanced ubiquitin chain binding and increased recruitment of OPTN to mitochondria [92], [115]. Moreover, it was found that OPTN interacts with TBK1, leading to recruitment of the kinase and its activation by autophosphorylation on damaged mitochondria [92], [115]. Similarly, TBK1 is recruited through its adaptor SINTBAD to NDP52 during antibacterial autophagy, and it is therefore present at the cargo from the early steps of autophagosome formation on Refs. [26], [32]. There it could phosphorylate OPTN to enhance its recruitment and to efficiently kill the bacteria by autophagy [30]. Increasing the affinity of cargo receptors for the cargo by phosphorylation might in turn increase the recruitment of the ULK1 complex to the cargo. The data summarized above suggest that TBK1 is a master regulator in this respect. Indeed, it was shown that NDP52-dependent mitophagy is defective in TBK1-deficient cells [24]. Furthermore, direct tethering of ULK1 to mitochondria leads to mitophagy also in TBK1-deficient cells and artificially tethering of TBK1 to mitochondria in the presence of ULK1/2 inhibitors fails to initiate mitophagy, suggesting that TBK1 acts upstream of ULK1 activation in NDP52 mediated autophagy [24].

Phosphorylation also plays an important role in enhancing the interaction of the ULK1/Atg1 complex with the cargo receptors. This is best understood for the yeast system, where the phosphorylation of the Atg19, Atg34, and Atg36 cargo receptors by Hrr25 is required for their function in delivering prApe1, Ams1, and peroxisomes to the vacuole. These phosphorylation events occur in a short motif and enhance the interaction of the receptors with the C-terminus of Atg11 [94], [116], [117]. Similar to the situation in yeast, the affinity of the interaction between the FIP200 claw domain and p62 is increased upon phosphorylation of four serine residues located in the vicinity of the p62 LIR domain [23]. Three of these residues are located in a short stretch of sequence similarity between p62 and Atg19 that contains the Hrr25 phosphorylation sites for Atg19 [23], [94]. The kinase responsible for p62 phosphorylation at these sites during the clearance of ubiquitinated proteins by selective autophagy remains to be identified.

Thus, a concept is emerging according to which ULK1/Atg1 recruitment and activation is possibly the most upstream event in the initiation of autophagosome formation. However, in starvation-induced bulk autophagy, its recruitment to the site of autophagosome formation may precede cargo recruitment and its activation is aided by global mTORC1 deactivation and perhaps by its clustering at the autophagosome formation site [48]. In selective, cargo-induced autophagy the cargo acts upstream via cargo receptors to recruit and cluster ULK1/Atg1 for its activation [23], [24], [25], [26], [28], [74].

## Outlook

In the past years, tremendous progress has been made regarding the recruitment and activation of ULK1/Atg1 during autophagy, in particular with regard to selective autophagy, where the cargo can be used as reference point to follow the assembly of the autophagy machinery. However, many questions regarding its recruitment and mechanisms of action still remain. Activation of the kinase complex is commonly associated with increased kinase activity. Indeed, ULK1/Atg1-mediated phosphorylation events are important drivers of autophagosome formation (reviewed in Refs. [40], [41]). On the other hand, the kinase domain makes up for only a fraction of the entire complex [118], and therefore, its role in setting the stage for productive autophagosome formation by acting as organizer and scaffold for autophagosome nucleation is likely to be equally important. For this reason, the activation of the complex may also entail the assembly and reorganization of the ULK1/Atg1 complex as well as its subunits at the site of autophagosome formation. This could not only bring the kinase domain in place to phosphorylate its targets but also to interact with and position the components of the autophagy machinery such as the PI3Kc1 [64], [65], ATG16L1 [66], [67], and Atg9 vesicles [42], [51], [119]. How these factors are positioned by the ULK1/Atg1 complex, how this positioning is linked to its kinase activity, and how these arrangements change during autophagosome formation, which is suggested by previous results [68], [120], are largely unclear. In all these events, the membrane source for autophagosome formation will also play an extremely important role because autophagosome formation cannot occur in the absence of lipids. It is currently unclear how the presence of the cargo and membrane recruitment are coordinated. However, it is possible that full activation of the autophagy machinery including the ULK1/Atg1 complex occurs only when the cargo and the donor membrane come into proximity as its activation may thereby be coupled to the presence of a membrane. Biochemically, the two surfaces provided by the cargo and the membrane might provide two high avidity binding platforms for full clustering and activation of the autophagy machinery.
